# Effectiveness of ω-3 Polyunsaturated Fatty Acids Based Lipid Emulsions for Treatment of Patients after Hepatectomy: A Prospective Clinical Trial

**DOI:** 10.3390/nu8060357

**Published:** 2016-06-17

**Authors:** Yuanfeng Gong, Zhaohui Liu, Yadi Liao, Cong Mai, Tiejun Chen, Hui Tang, Yunqiang Tang

**Affiliations:** 1 Department of Hepatobiliary Surgery, the Affiliated Cancer Hospital of Guangzhou Medical University, Guangzhou 510095, China; medgongyf@126.com (Y.G.) chenrf10@gmail.com (Y.L.); medmaic@126.com (C.M.); medchentj@126.com (T.C.); lzhzsdxsyx@gmail.com (H.T.); 2 Department of Anesthesiology, Cangzhou Central Hospital, Hebei Medical University, Cangzhou 061000, China; zhaohuiliu666@hotmail.com

**Keywords:** *n*-3 PUFAs, fish oil, Lipid emulsion, parenteral nutrition, hepatectomy

## Abstract

Objective: The present study aimed to investigate the effectiveness of parenteral nutritional support with ω-3 PUFAs–based lipid emulsions in patients after liver resection. Methods: A total of 119 patients were randomly assigned to the immunonutrition (IM) group (*n* = 59) and control group (*n* = 60). The IM group was continuously given Omegaven^®^ 10% 100 mL/day rather than regular nutrition for five days postoperatively. Venous blood samples were obtained from all subjects before surgery and D1, D3 and D7 after surgery. Results: No significant difference was found in baseline characteristics of the two groups. On D1 after surgery, no statistically significant differences were observed in the blood sample tests between the two groups. On D3 after surgery, the levels of white blood cell count (WBC), alanine aminotransferase (ALT), aspartate transaminase (AST) and total bilirubin (TBil) were dramatically decreased in the IM group (*t* = 3.065, *p* = 0.003; *t* = 2.149, *p* = 0.034; *t* = 5.313, *p*= 0.001; and *t* = 2.419, *p* = 0.017, respectively). Furthermore, on D7 after surgery, not only could a significant decrease be observed in the IM group concerning the levels of WBC, ALT and TBil (*t* = 3.025, *p* = 0.003; *t* = 2.094, *p* = 0.038; and *t* = 2.046, *p* = 0.043, respectively), but it was also seen in the level of Δprothrombintime (PT) (*t* = 2.450, *p* = 0.016). An increase in the level of prealbumin (Pre-Alb) in the IM group was observed on D7 after surgery (*t* = 2.237, *p* = 0.027). The frequency of total complications in the IM group were significantly lower than in the control group (χ^2^ = 4.225, *p* = 0.040 and χ^2^ = 3.174, *p* = 0.075). The trend favored the IM group in reducing the total infective complications rate (χ^2^ = 3.174, *p* = 0.075). A significant decrease in the duration of the hospital stay after surgery was also observed in the IM group (*t* = 2.012, *p* = 0.047).Conclusion: ω-3 PUFAs–based lipid emulsions for treatment of patients after hepatectomy are safe and effective in controlling inflammation, protecting liver function, and consequently reducing the rate of total complications and the duration of the hospital stay.

## 1. Introduction

Liver resection is the primary treatment for hepatic masses, especially malignant solid tumors, such as in liver cancer or liver metastasis. The prevalence of malnutrition is common among patients with advanced liver disease [1]. Perioperative nutritional support can significantly reduce the postoperative complications and/or mortality in patients with liver resection [2,3]. Considering liver dysfunction after ischemia-reperfusion injury, inflammatory response, nutrient metabolism abnormalities, and difference in liver cirrhosis status or resection range, nutritional support could be fraught with obstacles needing to be overcome. Therefore, postoperative nutritional support after liver resection must be individually and insightfully implemented.

Omega-3 polyunsaturated fatty acids (ω-3 PUFAs) are long-chain fatty acids including eicosapentaenoic acid (EPA), docosahexaenoic acid (DHA) and docosapentaenoic acid (DPA). Numerous studies have shown that parenteral nutritional support with ω-3 PUFAs–based lipid emulsions in critically ill patients could reduce the incidence of systemic inflammatory response syndrome (SIRS), the infection rate, and mortality [4,5]. Considering the severe surgical trauma, high rate of complications, and long time needed for recovery after liver resection, most patients need regular postoperative nutritional support, either parenteral nutrition (PN) or enteral nutrition (EN).

Thus, we performed this prospective clinical trial to comprehensively investigate the effectiveness of parenteral nutritional support with ω-3 PUFAs–based lipid emulsions in patients after liver resection.

## 2. Materials and Methods

### 2.1. Subjects

This prospective randomized clinical trial included 119 patients who received open hepatectomy between March 2011 and January 2016 at the Affiliated Cancer Hospital of Guangzhou Medical University. The study was approved by the Ethics Committee of the Hospital. Written informed consent was obtained directly from all participants. The diagnoses of liver cancer, liver metastasis or intrahepatic bile duct lithiasis were confirmed by postoperative pathological analysis. The exclusion criteria included patients treated with immunosuppressive drugs; plasma triglycerides concentration >400 mg/dL; chronic renal failure; acute angina or myocardial infarction; recent stroke; previous allergy to fish or egg proteins. One hundred and nineteen patients were randomized to the immunonutrition group (IM group) and the regular nutrition group (control group). The control group (regular nutrition group, *n* = 60) was given fat emulsion, amino acids (17AA) and glucose (11%) injection (Kabiven™ PI, Sino-Swed Pharmaceutical Co. Ltd, Jiangsu, China) 1920 mL/day via a central venous catheter. The supplementation started on the day after surgery and lasted 12 to 24 h. The IM group (*n* = 59) was continuously given Omegaven^®^10% 100 mL/day (Sino-Swed Pharmaceutical Co. Ltd, Jiangsu, China) every day, rather than regular nutrition. The non-protein calories were about 25 kcal/kg/day. Postoperative intravenous nutritional support lasted for five days. The 100 mL 10% Omegaven^®^ was injected into the package of Kabiven™ PI. Thus, patients were unable to ascertain the contents of nutrition.

Post-hepatectomy hemorrhage was defined as a drop in hemoglobin level >3 g/dL postoperatively compared with the baseline level and/or any postoperative transfusion of packed red blood cells for falling hemoglobin and/or the need for radiological intervention (such as embolization) and/or re-operation to stop bleeding. Evidence of intra-abdominal bleeding should be achieved by imaging or blood loss via the abdominal drains if present. Biliary leakage was defined as a drainage bilirubin to serum bilirubin ratio >3 at day 3 or later after resection or interventional surgical revision due to biliary peritonitis. Post-hepatectomy liver failure was defined as increased international normalized ratio and hyperbilirubinemia on or after postoperative day 5. A clinical diagnosis of sepsis was defined as systemic inflammatory response syndrome with a documented infection. Systemic inflammatory response syndrome was manifested by two or more of the following four variables: (1) hyperthermia >38.3 °C or hypothermia <36 °C; (2) tachycardia (>90 beats/min); (3) tachypnea (rate ≥ 20 breaths/min) or hypoxia (oxygen saturation <90% or need for oxygen supplementation of ≥0.4 fraction of inspired oxygen to maintain adequate saturation); and (4) leukocytosis (white blood cell count >12.0 × 10^9^/L), leukopenia (white blood cell count <4 × 10^9^/L), or immature or band forms >10%. Postoperative ileus was defined as two or more of nausea/vomiting, inability to tolerate oral diet over 24 h, absence of flatus over 24 h, distension, radiologic confirmation occurring on or after day 4 postoperatively without prior resolution of ileus.

White blood cell count (WBC) and C-reactive protein (CRP) were induced by the inflammatory reaction. Preoperative and postoperative liver function were assessed by Child-pugh score system based on serum alanine aminotransferase (ALT), aspartate transaminase (AST), total bilirubin (TBil), albumin (Alb), prealbumin (pre-Alb) and prothrombintime (PT) tests. Preoperative reserved liver function was measured by indocyanine green (ICG) 15 min clearance test. Serum triglyceride (TG) was used to measure fatty acid clearance. Venous blood samples were obtained from all subjects before surgery and one day (D1), three days (D3) and seven days (D7) after surgery. All patients’ preoperative characteristics (age, gender, BMI, Child-pugh score, and ICG15), intraoperative characteristics (resection range, blood loss, Pringle time, and surgery time) and postoperative characteristics (pathology, duration of postoperative hospital stay, complications and mortality) were collected.

### 2.2. Statistical Analysis

Mean and standard deviations are presented for continuous variables. Differences between the means of the two continuous variables were analyzed by the Student’s *t* test. To determine whether the frequencies between the IM group and control group were significantly different (α = 0.05), the χ*^2^* test was used. Statistical tests were two-sided and were considered statistically significant whenever *p* < 0.05. All analyses were performed by SPSS version 16.0 software (SPSS, Chicago, IL, USA).

## 3. Results

Concerning baseline characteristics of the two groups, no significant difference was found with respect to age, gender, BMI, Child-pugh score, ICG R15, pathology, resection range, blood loss, Pringle time or surgery time (Table 1). Also, blood sample tests of WBC, ALT, AST, TBil, Alb, Pre-Alb, TG and ΔPT between the two groups before surgery were not statistically different (Table 2).

On the first day after surgery, no statistically significant differences were observed in the above blood sample tests between the two groups. However, on the third day after surgery, the levels of WBC, ALT, AST and TBil were dramatically decreased in the IM group (*t* = 3.065, *p* = 0.003; *t* = 2.149, *p* = 0.034; *t* = 5.313, *p* = 0.001; and *t* = 2.419, *p* = 0.017, respectively). Furthermore, on the seventh day after surgery, not only could a significant decrease be observed in the IM group concerning the level of WBC, ALT and TBil (*t* = 3.025, *p* = 0.003; *t* = 2.094, *p* = 0.038; and *t* = 2.046, *p* = 0.043, respectively), but a decrease was also seen in the level of ΔPT (*t* = 2.450, *p* = 0.016). Although no significant differences were observed in the level of Alb, an increase in the level of Pre-Alb in the IM group was observed on the seventh day after surgery (*t* = 2.237, *p* = 0.027). No significant differences were observed in the level of TG (Table 2).

As shown in Table 3, the morbidity of total complications and total infection in the IM group were significantly lower than in the control group (χ^2^ = 4.225, *p* = 0.040). The trend favored the IM group in reducing the total infective complications rate (χ^2^ = 3.174, *p* = 0.075). A significant decrease in the duration of the hospital stay after surgery was also observed in the IM group (*t* = 2.012, *p* = 0.047). No significant difference was observed in the mortality between the two groups (Table 3).

## 4. Discussion

Clinical evidence showed that ω-3 PUFAs and other nutrients, such as immune glutamine, arginine, nucleosides and dietary fiber, could reduce excessive inflammatory reaction and modulate the immune response in cancer, surgical, and critically ill patients [6]. After liver resection, patients appear to have an increased level of aminotransferase, caused by surgical trauma, damage of liver cells and liver ultrastructure, and release of inflammatory mediators. Experimental studies have proved that ω-3 PUFAs effectively reduced severe hepatic steatosis and protected the liver from ischemia-reperfusion injury, which was associated with improved liver regeneration and functional recovery after hepatectomy [7].The present study showed that parenteral nutritional support with ω-3 PUFAs–based lipid emulsions after hepatectomy could dramatically reduce inflammation, protect liver function, and reduce the rate of total complications and the duration of the hospital stay.

The incorporation of PUFAs into the phospholipids of cell membranes ensures the maintenance of membrane fluidity and the adequate function of membrane proteins. Furthermore, ω-3 PUFAs exhibit strong anti-inflammatory and immunomodulatory effects via direct (by replacing arachidonic acid as an eicosanoid precursor) and indirect (by altering the expression of inflammatory genes through effects on transcription factor activation) mechanisms [8]. The ω-3/ω-6 PUFAs ratio released from the hydrolysis of membrane phospholipids influences the synthesis of eicosanoid mediators such as prostaglandins (PGs), thromboxanes (TXs), and leukotrienes (LTs). Eicosanoids (PGE2, TXA2, LT4) derived from ω-6 PUFAs show strong physiologic activities, such as leukocyte aggregation, taxis, and migration, and promote the synthesis of inflammatory cytokines and suppress cell-mediated immunity [8,9], while eicosanoids derived from ω-3 PUFAs stimulate peripheral monocytes and inhibit the synthesis of inflammatory cytokines IL-1 and TNF [10].

The recent discovery of families of novel pro-resolving lipid mediators that include the lipoxin, resolvin, protectin and maresin families has shed new light on the role of ω-3 PUFAs in the inflammatory process [11,12]. Pro-resolving lipid mediators derived from EPA are designated resolvins of the E series (RvE), while those derived from DHA are designated resolvins of the D series (RvD) [13]. These mediators have been shown to limit neutrophilic infiltration and enhance macrophage resolution responses, thus playing a role in diseases characterized by excessive uncontrolled inflammation [14,15,16]. Notably, their generation is related to the fatty acid composition of cellular membranes and can therefore be effectively increased with *n*-3 fatty acid supplementation [16].

Interestingly, in a mouse model system, researchers also found that supplementing the diet of tumor-bearing mice or rats with ω-3 PUFAs or inorganic selenium can slow the growth of various types of cancers [17,18]. A previous study highlighted the current knowledge of the potential mechanisms of the anti-carcinogenic actions of ω-3 fatty acids. These included suppression of arachidonic acid–derived eicosanoid biosynthesis; influence on transcription factor activity, gene expression, and signal transduction; alteration of estrogen metabolism; increased or decreased production of free radicals and reactive oxygen species; and effect on insulin sensitivity and membrane fluidity [19].

In the present study, on the third day after surgery, the level of WBC was dramatically decreased in the IM group, suggesting that ω-3 PUFAs–based parenteral nutritional support could suppress an excessive inflammatory reaction after liver resection. High sensitive C-reactive protein (hsCRP) is a more sensitive index than WBC, revealing inflammatory reactions in the human body. Unfortunately, hsCRP was not mainstreamed in our institution. ALT, AST and TBil were dramatically decreased in the IM group, while Pre-Alb was increased in the IM group, revealing that early postoperative implementation of ω-3 PUFAs–based parenteral nutritional support can be beneficial for liver function recovering from ischemia-reperfusion injury after hepatectomy. The conclusion was consistent with a previous study, which showed that fish oil–based emulsion is hepatoprotective in a murine model of parenteral nutrition–associated liver disease, and it appears to be safe and effective for the treatment of this type of liver disease in children [20]. Although the rate of total infective complications was not significantly different between the two groups, the trend favored the IM group in reducing the rate of infective complications. Furthermore, the rates of total complications and the duration of the hospital stay were greatly decreased in the IM group. The result was consistent with previous studies where ω-3 PUFAs–based lipid emulsions were implemented in critically ill patients or those who underwent gastrointestinal surgery [21].

In summary, the present study suggested that ω-3 PUFAs–based lipid emulsions for treatment of patients after hepatectomy re safe and effective in controlling inflammation, protecting liver function, and consequently reducing the rate of total complications and the duration of the hospital stay. These findings add new evidence to the clinical practice of parenteral nutritional support with ω-3 PUFAs–based lipid emulsions implemented after hepatectomy. Given the small sample size included in the present studies, further prospective multicenter studies with larger sample sizes and good control for confounding factors are needed to affirm the effect of ω-3 PUFAs–based lipid emulsions in patients after hepatectomy.

## Figures and Tables

**Table 1:** Baseline characteristics of the two groups.

	IM Group (%) (*n* = 59)	Control Group (%) (*n* = 60)	*t*/χ^2^	*p* Value
Age (year)	51.37 ± 11.75	49.63 ± 11.21	0.827	0.410
Gender				
Male	44 (74.6)	46 (76.7)	0.071	0.791
Female	15 (25.4)	14 (23.3)		
BMI				
≤18.5	10 (16.9)	8 (13.3)	0.579	0.901
18.5–22.9	23 (39.0)	22 (36.7)		
23.0–24.9	21 (35.6)	25 (41.7)		
>25	5 (8.5)	5 (8.3)		
FBG (mmol/L)	5.3 ± 1.7	4.7 ± 2.1	1.711	0.090
HBsAg positive	56 (94.9)	58 (96.7)	0.001	0.985
HBV-DNA ≥ 1 × 10^3^ (copies/mL)	49 (83.1)	47 (78.3)	0.425	0.515
Child-pugh score				
Score A	53 (89.8)	52 (86.7)	0.287	0.592
Score B	6 (10.2)	8 (13.3)		
ICG R15 (%)	8.43 ± 5.94	7.62 ± 4.79	0.820	0.414
Pathology				
Liver cancer	53 (89.8)	56 (93.3)	0.557	0.757
Liver metastases	4 (6.8)	3 (5.0)		
Benign diseases	2 (3.4)	1 (16.7)		
Resection range				
1 segment	11 (18.6)	9 (15.0)	0.516	0.915
2 segments	24 (40.7)	23 (38.3)		
3 segments	18 (30.5)	21 (35.0)		
≥4 segments	6 (10.2)	7 (11.7)		
Blood loss (mL)	423.3 ± 395.8	451.4 ± 243.3	0.467	0.641
Pringle time (min)	18.6 ± 12.0	16.7 ± 9.2	0.970	0.334
Surgery time (min)	267.9 ± 112.0	289.4 ± 147.5	0.894	0.373

FBG, fasting blood glucose; IM, immunonutrition; BMI, body mass index; ICG R15, indocyanine green retention at 15 min.

**Table 2:** Blood sample tests of the two groups before and after surgery.

	IM Group (%) (*n* = 59)	Control Group (%) (*n* = 60)	*t*	*p* Value
WBC (×10^9^/L)				
Preoperation	7.24 ± 2.65	6.59. ± 3.14	1.219	0.225
D1	16.48 ± 6.17	17.69 ± 5.84	1.099	0.274
D3	10.47 ± 3.51	12.82 ± 4.75	3.065	0.003
D7	8.26 ± 3.29	9.92 ± 2.67	3.025	0.003
ALT (IU/L)				
Preoperation	35.47 ± 18.92	36.19 ± 14.62	0.233	0.817
D1	351.36 ± 239.17	417.94 ± 274.52	1.410	0.161
D3	194.64 ± 76.82	237.84 ± 134.27	2.149	0.034
D7	54.63 ± 27.28	63.67 ± 34.90	2.094	0.038
AST (IU/L)				
Preoperation	34.50 ± 14.97	35.83 ± 16.20	0.465	0.643
D1	394.35 ± 197.03	463.16 ± 257.38	1.636	0.105
D3	152.28 ± 89.61	268.20 ± 142.09	5.313	0.001
D7	41.23 ± 35.19	52.01 ± 37.20	1.623	0.107
TBil (umol/L)				
Preoperation	10.63 ± 4.71	11.93 ± 5.29	1.415	0.160
D1	26.37 ± 9.45	28.64 ± 11.64	1.167	0.246
D3	17.09 ± 4.94	19.34 ± 5.20	2.419	0.017
D7	14.53 ± 4.47	16.46 ± 5.73	2.046	0.043
Alb (g/L)				
Preoperation	38.71 ± 12.30	39.68 ± 11.61	0.443	0.659
D1	28.13 ± 9.97	26.64 ± 8.46	0.880	0.381
D3	33.78 ± 8.87	32.47 ± 7.69	0.861	0.391
D7	38.76 ± 9.67	37.61 ± 8.94	0.674	0.502
Pre-Alb (mg/L)				
Preoperation	131.37 ± 51.75	139.63 ± 47.21	0.910	0.365
D1	97.63 ± 63.71	86.71 ± 42.97	1.098	0.275
D3	124.67 ± 71.97.	108.07 ± 56.97	1.396	0.165
D7	159.76 ± 85.28	129.24 ± 61.90	2.237	0.027
TG (mmol/L)				
Preoperation	0.79 ± 0.19	0.86 ± 0.21	1.906	0.059
D1	1.24 ± 0.28	1.21 ± 0.24	0.628	0.531
D3	1.18 ± 0.21	1.14 ± 0.26	0.922	0.358
D7	0.85 ± 0.17	0.91 ± 0.27	1.448	0.150
ΔPT(s)				
D1	0.73 ± 0.95	0.84 ± 1.12	0.577	0.565
D3	0.67 ± 0.89	0.78 ± 0.98	0.641	0.523
D7	0.26 ± 0.19	0.35 ± 0.21	2.450	0.016

IM, immunonutrition; D, day; WBC, white blood cell count; CRP, C-reactive protein; ALT, alanine aminotransferase; AST, aspartate transaminase; TBil, total bilirubin; Alb, albumin; Pre-Alb, prealbumin; TG, triglyceride; PT, prothrombin time.

**Table 3:** Complications, mortality, and duration of hospital stay after surgery between two groups.

	IM Group (%) (*n* = 59)	Control Group (%) (*n* = 60)	χ^2^/*t*	*p* Value
Total complications	15 (25.4)	26 (41.7)	4.225	0.040
Abdominal hemorrhage	1	1	0.000	1.000 ^b^
Upper gastrointestinal hemorrhage	1	2	0.000	1.000 ^b^
Biliary leakage	2	3	0.000	1.000 ^b^
Liver failure	1	2	0.000	1.000 ^b^
Total infection	8 (13.6)	16 (25.0)	3.174	0.075
Wound infection	2	3	0.000	1.000 ^b^
Pulmonary infection	3	6	0.445	0.505 ^b^
Abdominal infection	1	3	0.242	0.623 ^b^
Urinary tractinfection	2	4	0.158	0.691 ^b^
Sepsis	1	3	0.242	0.623 ^b^
ileus	2	2	0.000	1.000 ^b^
Mortality	0 (0)	1 (0.02)		1.000 ^a^
Duration of hospital stay after surgery	8.47 ± 5.36	10.56 ± 6.95	2.012	0.047

IM, immunonutrition; ^a^ Fisher’s exact test; ^b^ continuity correction.
